# Automated Spectral Preprocessing via Bayesian Optimization for Chemometric Analysis of Milk Constituents

**DOI:** 10.3390/foods14172996

**Published:** 2025-08-27

**Authors:** Habeeb Abolaji Babatunde, Owen M. McDougal, Timothy Andersen

**Affiliations:** 1 Computer Science, Boise State University, Boise, ID 83725, USA; habeebbabatunde@u.boisestate.edu; 2 Department of Chemistry and Biochemistry, Boise State University, Boise, ID 83725, USA; owenmcdougal@boisestate.edu

**Keywords:** spectral preprocessing, Bayesian optimization, milk, chemometrics, infrared spectroscopy, PLS, machine learning, dairy, food, regression analysis

## Abstract

The preprocessing of infrared spectra can significantly improve predictive accuracy for protein, carbohydrate, lipid, or other nutrition components, yet optimal preprocessing selection is typically empirical, tedious, and dataset specific. This study introduces a Bayesian optimization-based framework designed for the automated selection of optimal spectral preprocessing pipelines within a chemometric modeling context. The framework was applied to mid-infrared spectra of milk to predict compositional parameters for fat, protein, lactose, and total solids. A total of 385 averaged spectra corresponding to 198 unique samples was split into a 70/30 ratio (training/test) using a group-aware Kennard-Stone algorithm, resulting in 269 averaged spectra (135 unique samples) for training and 116 spectra (58 unique samples) for testing. Six regression models: Elastic Net, Gradient Boosting Machines (GBM), Partial Least Squares (PLS), RidgeCV Regression, LassoLarsCV, and Support Vector Regression (SVR) were evaluated across three preprocessing conditions: (1) no preprocessing, (2) literature-derived custom preprocessing (e.g., MSC, SNV, and first and second derivatives), and (3) optimized preprocessing via the proposed Bayesian framework. Optimized preprocessing consistently outperformed other methods, with RidgeCV achieving the best performance for all components except lactose, where PLS slightly outperformed it. Improvements in predictive accuracy, particularly in terms of RMSEP were observed across all milk components. The best RMSEP results were achieved for protein (RMSEP = 0.054, $R^{2}=0.981$) and lactose (RMSEP = 0.026, $R^{2}=0.917$), followed by fat (RMSEP = 0.139, $R^{2}=0.926$) and total solids (RMSEP = 0.154, $R^{2}=0.960$). Literature-based pipelines demonstrated inconsistent effectiveness, highlighting the limitations of transferring preprocessing methods between datasets. The Bayesian optimization approach identified relatively simple yet highly effective preprocessing pipelines, typically involving few steps. By eliminating manual trial and error, this data-driven strategy offers a robust and generalizable solution that streamlines spectral modeling in dairy analysis and can be readily applied to other types of spectroscopic data across various domains.

## 1. Introduction

Milk is a nutrient-dense biological fluid that contains essential macronutrients, such as fat, protein, and lactose, along with vitamins, minerals, and bioactive compounds that support human growth, health, and disease prevention [1]. Fats contribute to energy provision, flavor, and fat-soluble vitamin transport; proteins such as casein and whey not only supply essential amino acids with high biological value but also exert a range of bioactive functions, including antibacterial, immunomodulatory, antioxidant, antihypertensive, and opioid-like activities, in addition to providing functional properties important in dairy processing [2]. Lactose serves as an energy source and facilitates calcium absorption, while minerals such as calcium, phosphorus, and magnesium are critical for bone development and metabolic functions [2]. The balance of these constituents determines not only the nutritional value of milk but also its technological functionality in the production of a wide range of dairy products [2]. The accurate determination of these components is therefore fundamental for quality control, economic valuation, and optimization of dairy production.

Conventional analysis of milk composition relies on standardized wet-chemical and instrumental reference methods to ensure accuracy and compliance. Protein content is determined using Kjeldahl nitrogen analysis or Dumas combustion; fat is measured through Gerber/Babcock acid digestion or Rose–Gottlieb solvent extraction; lactose is quantified by polarimetry; and minerals are analyzed through ashing followed by atomic absorption spectroscopy (AAS) or inductively coupled plasma (ICP) spectroscopy [3,4,5,6,7]. High-performance liquid chromatography (HPLC) is a versatile technique that can be applied for the quantification of lactose, proteins, fats, vitamins, and other bioactive compounds, offering high sensitivity and specificity across multiple milk components. While these conventional methods deliver high precision, they are often labor intensive, time consuming, and require skilled operators, making them less suitable for real-time process control in modern dairy operations [8].

In recent years, advanced food processing technologies, such as high-pressure processing (HPP), microfiltration, pulsed electric fields (PEFs), UV-C (ultraviolet C) treatment, and high-pressure homogenization, have been developed to enhance microbial safety, extend shelf life, and preserve the nutritional and sensory qualities of milk [9]. HPP inactivates pathogenic and spoilage microorganisms using a hydrostatic pressure of 400–600 MPa without significant heat, maintaining vitamins, flavor, and protein functionality [10]. Microfiltration employs membrane separation to remove bacteria, spores, and somatic cells while retaining desirable components such as proteins and minerals [11]. PEF uses short bursts of high-voltage electric pulses to disrupt microbial cell membranes, achieving pasteurization-like safety with minimal thermal damage [12]. UV-C treatment, operating in the 200–280 nm wavelength range, inactivates microbes by damaging their DNA [13]. It is particularly effective for surface decontamination and thin-film liquid applications, helping preserve the nutritional and sensory qualities of foods while extending shelf life [14]. High-pressure homogenization applies intense shear forces at elevated pressures to reduce fat globule size, improve emulsion stability, and enhance microbial inactivation [15]. Complementing these advances, rapid analytical tools, including vibrational spectroscopies (mid-infrared, near-infrared, and Raman), fluorescence sensors, dielectric/impedance detectors, and biosensors, enable non-destructive, on-site measurement of key components and contaminants within seconds. These analytical systems can function independently or in tandem with processing methods to verify composition, confirm microbial inactivation, detect adulteration, and optimize parameters in real time. Their integration, particularly through inline sensors and IoT-enabled monitoring, closes the loop between processing and quality assurance, ensuring milk safety, consistency, and consumer acceptability.

Mid-infrared (MIR) spectroscopy, when coupled with chemometric modeling, offers a rapid, non-destructive, and cost-effective alternative to improve predictive accuracy and processing efficiency [8,16]. MIR spectroscopy measures the absorption of infrared radiation within the mid-infrared region of the electromagnetic spectrum from 4000–400 cm^−1^ spectral range, where molecular vibrations associated with specific functional groups occur [17]. These vibrations correspond to the stretching and bending motions of chemical bonds, making MIR highly effective for identifying and quantifying key milk components. For instance, in the MIR spectral range of 4000 to 400 cm^−1^, various absorption peaks correspond to the vibrational modes of molecular bonds in milk components interacting with infrared radiation [18]. Fats are characterized by absorption bands associated with the stretching vibrations of C-H bonds in fatty acid chains. In particular, peaks at approximately 2922 cm^−1^ and 2852 cm^−1^ correspond to the asymmetric and symmetric stretching vibrations of the methylene (CH_2_) groups, respectively. Additionally, an absorption peak around 1743 cm^−1^ is linked to the C=O stretching vibrations of ester carbonyl groups in triglycerides, providing a distinctive marker for lipids in milk [18]. The spectral range between 1700 cm^−1^ and 1500 cm^−1^ is characterized by prominent peaks associated with peptide bonds in proteins. Two major bands are the amide I band around 1635 cm^−1^, attributed to C=O stretching and N-H bending vibrations, and the amide II band near 1548 cm^−1^, corresponding to N-H bending coupled with C-N stretching. These bands are directly related to the peptide bonds in milk proteins such as casein and whey proteins [19]. Additionally, the region between 1200 and 900 cm^−1^ contains absorption peaks linked to carbohydrates, particularly lactose. For instance, a peak at approximately 1077 cm^−1^ is associated with C-O stretching vibrations in lactose [20].

Despite these advantages, MIR spectra are inherently convoluted due to overlapping absorption bands from various constituents. Unlike HPLC, which yields distinct peaks for individual analytes, MIR does not allow the direct deconvolution of each component without additional statistical modeling. As a result, chemometrics plays a crucial role in the deconvolution of MIR spectra and linking them to quantifiable milk components such as fat, protein, lactose, and total solids [8]. A typical chemometric workflow includes three essential steps: spectral preprocessing, wavenumber selection, and predictive model development. Of these, preprocessing is foundational because the raw spectra often contain instrumental noise, baseline drift, scattering effects, and sample inconsistencies that obscure meaningful chemical information [21]. Spectral preprocessing comprises mathematical transformations designed to minimize unwanted variation and enhance relevant features of the spectra. Common methods include baseline correction, scatter correction (e.g., standard normal variate (SNV), multiplicative scatter correction (MSC)), smoothing, normalization, and derivatives (e.g., Savitzky–Golay (SavGol)) [22]. These techniques improve the signal-to-noise ratio and promote consistency across samples, thus enhancing the accuracy and robustness of the resulting chemometric models [21,23]. However, choosing the appropriate preprocessing pipeline remains a significant challenge. Most studies rely on manual selection or predefined methods from previous work, often without evaluating their suitability for the current dataset or target analyte [22,24]. This trial-and-error approach introduces subjectivity and can lead to suboptimal model performance. Several studies have highlighted the limitations of such practices. For example, Zhu et al. [25] demonstrated that transferring preprocessing techniques such as SNV, SavGol, and first- and second-order derivatives developed for fruit ripening [26], portable near-infrared (NIR) devices for milk assessment [27], and meat quality classification [28] to dielectric spectroscopy for milk fat analysis yielded poor calibration performance, with only SNV in combination with least squares support vector machines (LSSVMs) providing marginal gains. Pinto et al. [29] similarly showed that preprocessing effectiveness in MIR-based lactose prediction depended heavily on the selected spectral region and transformation method. Amsaraj et al. [30] applied preprocessing pipelines derived from tea sample analysis to milk adulterant detection with limited success, underscoring the risks of direct method transfer. Inon et al. [31] observed that the application of MSC, originally developed for NIR spectra, failed to improve the prediction accuracy when adapted to FTIR spectra. Collectively, these works reveal the necessity of dataset-specific preprocessing optimization.

Bayesian optimization (BO) offers a principled framework for addressing this issue. Unlike grid or random search methods, which either exhaustively or blindly explore the hyperparameter space, BO employs probabilistic models (e.g., Gaussian processes or Tree-structured Parzen Estimators) to guide the search toward promising regions of the solution space [32,33]. This enables efficient and scalable optimization, particularly in high-dimensional or complex domains like spectral preprocessing. In chemometrics, BO has been shown to outperform greedy or uninformed strategies in tasks such as PLS calibration [33], spectral feature selection [34], and MIR-based protein quantification [8].

Despite recent advances in wavenumber selection and model tuning, preprocessing optimization remains underexplored. Notable efforts such as the nippy package by Torniainen et al. [22] introduced the automated comparison of preprocessing strategies but relied on greedy search, which becomes computationally expensive and lacks global exploration capabilities. Moreover, the need for more adaptive and scalable preprocessing optimization has been emphasized in the recent chemometric literature [21,22,24].

Motivated by this gap, we propose a novel framework for automated preprocessing optimization in spectroscopic data analysis. The approach integrates spectroscopy-specific and general machine learning preprocessing techniques and leverages Gaussian Process-based Bayesian Optimization to dynamically identify the most effective pipeline for each predictive task. We apply this method to a mid-infrared (MIR) milk spectroscopy dataset to optimize the prediction of fat, protein, lactose, and total solids. Our results demonstrate that data-driven preprocessing selection within a chemometric modeling framework improves model accuracy and robustness while reducing the reliance on intuition and manual tuning. Although developed for milk analysis, this framework is a generalizable solution applicable to a wide range of infrared spectroscopic datasets in food, pharmaceutical, environmental, and agricultural domains.

## 2. Materials and Methods

### 2.1. Spectra Acquisition

MIR spectral data of milk were obtained from Agropur Jerome Cheese, Jerome, ID, USA; the dataset consisted of MIR spectra obtained during routine milk analysis during processing. All spectra were collected using a MilkoScan FT1 (Foss North America, Eden Prairie, MN, USA). The MIR dataset included samples from multiple production sources, encompassing various vats, raw tanks (RT), and other operational milk streams that are sampled throughout everyday operations. All spectra were provided in their raw form (absorbance units), without any additional preprocessing applied prior to analysis.

### 2.2. Dataset Description

The dataset used in this study comprises a total of 1772 spectral and 1193 reference data. After aligning and matching the two sources, 6362 spectral-reference matched samples were obtained. Each spectrum consists of 1060 variables, corresponding to wavenumbers ranging from 4999.99 cm^−1^ to 925.07 cm^−1^, with an approximate step size of 4 cm^−1^. These spectra were paired with the 4 target variables used in this study: fat (%), true protein (%), lactose (%), and total solids (TS, %). Due to multiple replicates within the spectral data (e.g., samples labeled as VAT15_1, VAT15_2, with repeated measurements such as VAT15_1, VAT15_1, etc.), a data reduction step was necessary. To resolve redundancy and improve consistency, mean spectra were computed for replicates of each unique sample group, resulting in a final dataset containing 385 averaged spectra and 193 unique samples.

### 2.3. Data Splitting

To ensure robust model validation, a modified Kennard-Stone algorithm with replicate handling (Kennard-StoneR) was implemented for data partitioning as presented in Algorithm 1. This approach addresses a critical limitation in the vanilla Kennard-Stone method [35], which can inadvertently place replicates from the same sample in both training and test sets, potentially leading to data leakage and overly optimistic model performance estimates. The Kennard-StoneR algorithm maintains the core principle of the original Kennard-Stone method that maximizes the Euclidean distance between selected samples to ensure representative coverage of the feature space while incorporating group-aware selection to prevent replicate splitting. The algorithm proceeds as follows.
**Algorithm 1** Modified Kennard-Stone with replicate handling (Kennard-StoneR).**Require:** Data matrix $X\in R^{n\times d}$, labels $y\in R^{n}$, group identifiers $g\in R^{n}$, test proportion *p*
**Ensure:** Training and test indices
1:**Aggregate replicates** by computing the centroid of each sample group:2:**for** each unique group identifier *i* **do**3:      ${\bar{X}}_{i}\leftarrow \mathrm{mean}\left(X\left[g=i\right]\right)$4:**end for**5:**Determine** the number of training groups:6:m←round((1−p)×numberofuniquegroups)7:**Compute** the distance matrix D between all group centroids:8:$D_{ij}\leftarrow {∥{\bar{X}}_{i}-{\bar{X}}_{j}∥}_{2}$9:**Initialize** selected groups *S* with the pair of groups having maximum distance:10:$S\leftarrow \{\arg\max_{\left(i,j\right)}D_{ij}\}$11:**while** |S|<m **do**12:    For each unselected group *u*:13:       Compute minimum distance to any selected group:14:       $d_{\min}\left(u\right)\leftarrow \min\left\{D_{su}:s\in S\right\}$15:    Add the unselected group with maximum minimum distance:16:    $S\leftarrow S\cup \{\arg\max_{u}d_{\min}\left(u\right)\}$17:**end while**18:**Map** selected groups to original sample indices:19:train_indices ←{j:g[j]∈S}20:test_indices ←{j:g[j]∉S}21:**return X**[train_indices], **X**[test_indices], **y**[train_indices], **y**[test_indices]


The algorithm generates a training set that optimally spans the feature space while reserving a representative portion (p) of samples for independent testing. For our 193 unique milk composition MIR spectra, this technique resulted in a training set comprising 70% or 135 unique samples and a test set with the remaining 30% or 58 samples, with the complete separation of replicates between sets. Group-aware cross-validation was implemented by grouping samples that share the same base identifier, regardless of replicate index. For example, samples labeled VAT15_1 and VAT15_2 were treated as belonging to the same group (VAT15). This approach ensured that all replicates of a given sample were kept together during both training and testing, thereby preventing data leakage. In total, 193 unique groups were identified based on this naming convention. We verified that spectra within each group had similar reference (target) values, confirming their validity as true replicates.

### 2.4. Automated Pipeline Optimization for Spectral Preprocessing and Modeling

To enhance the robustness, reproducibility, and efficiency of spectral data analysis, we developed a Python-based framework for automated preprocessing pipeline optimization. At its core is the PipelineOptimizer class, which leverages Bayesian optimization [36] to systematically explore and fine-tune combinations of preprocessing techniques and model hyperparameters. This process is designed to yield the most predictive and scientifically valid pipeline tailored to the user’s dataset.

**Figure 1:**
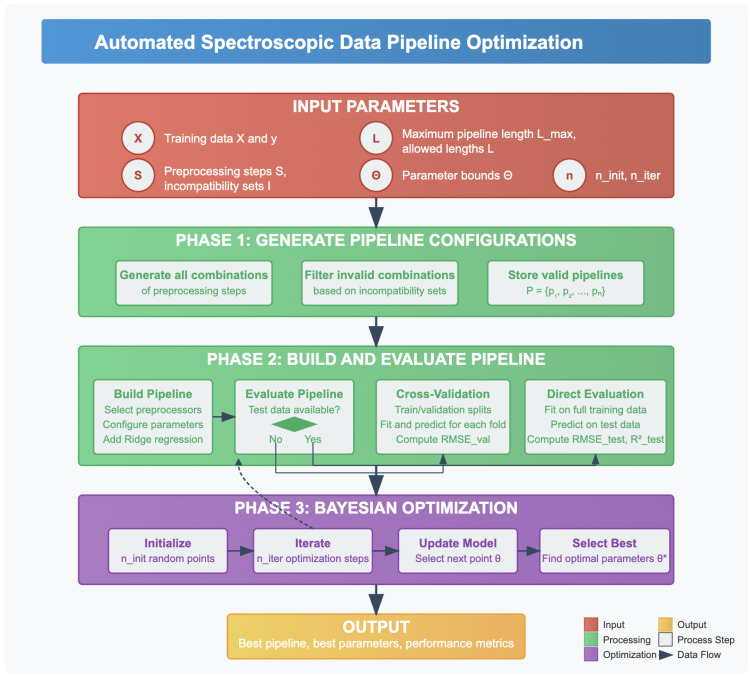
Automated spectroscopic data pipeline optimization framework. The workflow integrates preprocessing, evaluation, and Bayesian optimization to discover optimal preprocessing sequences. Phase 1 generates valid pipeline configurations based on compatibility constraints. Phase 2 evaluates pipelines through cross-validation or direct test set assessment. Phase 3 applies Bayesian optimization to fine-tune hyperparameters, ultimately yielding preprocessing pipelines that maximize model performance for spectroscopic data analysis.

The framework supports a diverse set of preprocessing methods, including both spectroscopy-specific transformations and general purpose machine learning preprocessing from scikit-learn. During optimization, the framework intelligently excludes incompatible combinations based on predefined rules as described subsequently, ensuring that only valid configurations are evaluated. The high-level workflow, encompassing preprocessing configuration, validation, and pipeline optimization, is summarized in Algorithm 2 and Figure 1. A complete version with detailed steps and procedures is provided in Appendix A (Algorithm A1). This algorithm outlines the core logic behind candidate generation, evaluation using cross-validation or test data, and the Bayesian search strategy employed for optimization. This structured and reproducible approach provides a powerful tool for advancing chemometric analysis in both research and applied settings.
**Algorithm 2** Automated spectroscopic data pipeline optimization.1:**Input:** $X_{train}$, $y_{train}$, preprocessing steps S, incompatibilities I, allowed lengths L, bounds Θ, $n_{init}$, $n_{iter}$2:**Optional:** $X_{test}$, $y_{test}$3:**procedure** GeneratePipelines (S,I,L)4:    Generate all valid preprocessing pipelines P subject to incompatibilities5:**end procedure**6:**procedure** Evaluate(θ)7:    Decode θ to build pipeline $p_{\theta }$8:    **if** $X_{test}$ is available **then**9:        Fit and evaluate $p_{\theta }$ on test set10:    **else**11:        Cross-validate $p_{\theta }$ on training set12:    **end if**13:    **return** negative RMSE as score14:**end procedure**15:**procedure** Optimize($\Theta ,n_{init},n_{iter}$)16:    Use Bayesian optimization to find $\theta^{*}$ maximizing Evaluate17:    Build best pipeline $p^{*}$ from $\theta^{*}$18:    Fit $p^{*}$ on $X_{train}$; evaluate on $X_{test}$ if available19:    **return** $p^{*}$, $\theta^{*}$20:**end procedure**21:**Output:** Optimized pipeline $p^{*}$ and parameters $\theta^{*}$

#### 2.4.1. Overview of Pipeline Optimization Strategy

The PipelineOptimizer class supports spectral datasets formatted as NumPy arrays, allowing users to specify training and testing sets, cross-validation strategies, and optional grouping variables. The framework incorporates two group-aware validation strategies: GroupShuffleSplit and LeavePGroupsOut, ensuring the robust evaluation of pipelines in the presence of samples with repeated measurements [37,38].

#### 2.4.2. Preprocessing Configuration Space

Users can provide a custom list of candidate preprocessing steps which are then filtered for compatibility using a set of predefined rules. The framework supports the following spectroscopy-specific preprocessing methods: SNV, SavGol, MSC, Extended Multiplicative Signal Correction (EMSC), Mean Centering (MeanCN), Detrending, AsymmetricLeastSquareBaselineCorrection, Localized SNV (LSNV), and Robust Normal Variate (RNV) [39,40,41]. Additionally, the framework seamlessly integrates general purpose preprocessing methods from scikit-learn: Standard Scaling, Robust Scaling, Global Scaling, MinMaxScaler, Normalization, QuantileTransformer, Principal Component Analysis (PCA), Locally Linear Embedding (LLE), fast Independent Component Analysis (fast-ICA), kernel-PCA, and PowerTransformer. All valid preprocessing pipelines comprising up to a user-defined maximum number of steps are enumerated in advance, allowing optimization to occur over this discrete configuration space. Users can further constrain the search by specifying the allowed pipeline lengths. For example, setting the maximum pipeline length to 2 allows for either single preprocessing steps or combinations of two compatible methods.

#### 2.4.3. Bayesian Optimization

Pipeline optimization is performed using Bayesian optimization with an Expected Improvement (EI) acquisition function via the Bayesian optimization Python library to autonomously identify optimal preprocessing pipelines, thereby eliminating the traditionally labor-intensive process of manual tuning in spectroscopic analysis [36]. Bayesian optimization is a probabilistic model-based approach that efficiently locates the extrema of objective functions with minimal evaluations, making it particularly well-suited for complex optimization tasks [36]. The optimization process begins with $n_{init}$ random initial configurations, followed by $n_{iter}$ intelligently selected configurations guided by the Bayesian model’s posterior distribution (with both $n_{init}$ and $n_{iter}$ defined by the user). The objective function dynamically constructs and evaluates pipelines based on a sampled index into the list of possible preprocessing configurations. Each pipeline is appended with a Ridge regression estimator, where the regularization strength (θ) is also optimized.

During each evaluation, the selected pipeline is fitted and validated using the configured cross-validation strategy. The objective function returns the negative Root Mean Squared Error (RMSE), penalizing unstable or ill-conditioned configurations (e.g., those leading to LinAlgError). Logging is integrated throughout the optimization process to track evaluated configurations, metric values (RMSE, $R^{2}$), and potential numerical issues.

#### 2.4.4. Cross-Validation Methods

To ensure robust and realistic evaluation of preprocessing pipelines, we implemented group-aware cross-validation strategies suitable for spectroscopic data and chemometric modeling. These methods are particularly suited for spectral datasets, where measurements may be recorded as replicates. The framework allows users to provide an optional group parameter, specifying the group to which each sample belongs. If no group information is supplied, each sample is treated as independent, and traditional sample-level validation is performed. Two primary group-based cross-validation techniques are supported:

1. Group-Shuffle-Split: This method randomly divides groups of samples into training and validation sets while ensuring that all samples within a group are assigned to the same split. This technique helps mitigate data leakage and preserves the natural structure of the data, which is important for spectral datasets prone to replicate effects.
2. Leave-P-Groups-Out: This exhaustive method iteratively leaves out *P* groups as a validation set, training on the remaining groups. It offers a more stringent assessment of generalization to unseen groups, though at a higher computational cost.

In addition to these cross-validation strategies, we enhanced the evaluation function with conditional logic to leverage external test data when available. Specifically, we added a mechanism to check whether both X_test and y_test are present. If test data is provided, the evaluation proceeds as follows:

- The pipeline is fit on the training data.
- Predictions are made on the external test data.
- Performance metrics: Root Mean Squared Error (RMSE) and coefficient of determination ($R^{2}$) are computed on the test set.
- The negative RMSE is returned as the optimization score for compatibility with minimization-based search frameworks.

If external test data is not available, or if an error occurs during test-based evaluation, the function defaults to the original group-based cross-validation strategy using either Group-Shuffle-Split or Leave-P-Groups-Out.

#### 2.4.5. Compatibility Rules for Preprocessing Pipelines

The framework is designed to support a wide range of preprocessing techniques, drawing from both domain-specific spectroscopic methods and general purpose machine learning transformations available through scikit-learn. While this flexibility enables the construction of diverse and powerful pipelines, it also introduces the risk of combining methods that are theoretically redundant, semantically incompatible, or computationally conflicting.

To address this, we implemented a set of incompatibility rules that automatically prevent mutually exclusive or conceptually redundant methods from being used together. These rules are defined based on both functional similarity and insights from prior spectroscopic and chemometric literature [22].

For example, the following groups of preprocessing steps are treated as mutually incompatible:

- Scatter Correction Methods: SNV, MSC, EMSC, LSNV, RNV are all methods that correct for scatter effects in spectral data. Applying more than one of these techniques can lead to overcorrection or unintended distortions.
- Scaling and Normalization Methods: Methods such as scaler, autoscale, globalscale, normalization, robust-scaler, minmax-scaler, power-transformer, quantile-transformer, and row-standardizer all perform some form of scaling or normalization. Using multiple scaling approaches in the same pipeline may introduce redundancy and instability.
- Method-Specific Incompatibilities: Specific combinations such as SNV with row-standardizer, or autoscale with scaler, are excluded due to their overlapping functionalities.
- Dimensionality Reduction Methods: Techniques such as PCA, fast-ICA, kernel-PCA, and LLE aim to reduce data dimensionality and are typically not applied together, as they each represent distinct reduction philosophies.

These constraints are enforced internally through a predefined list of incompatibility sets. When a user supplies a list of candidate preprocessing techniques, some of which may be mutually incompatible, the framework ensures that such combinations are automatically excluded from consideration during pipeline optimization. Instead of raising an error, the system filters out any configurations that violate the defined compatibility rules, thereby streamlining the search space and maintaining both computational efficiency and methodological validity. This ensures that only scientifically coherent and practically feasible pipelines are explored during the optimization process, in line with established best practices in chemometric data preprocessing [22].

By integrating these methodological advances, the proposed framework represents a significant improvement over traditional approaches to spectroscopic data preprocessing, enabling more systematic, objective, and reproducible preprocessing pipeline optimization for chemometric applications.

### 2.5. Regression Analysis

To model the relationship between spectral features and the target variable(s), we employed six regression algorithms provided by the scikit-learn library [42]. These included Elastic Net, Partial Least Squares (PLS), Support Vector Regression (SVR), LassoLarsCV, RidgeCV, and Gradient Boosting Machines (GBMs).

The regression models are briefly described below:

- **Elastic Net** regression combines both L1 (Lasso) and L2 (Ridge) regularization penalties. It is particularly effective for datasets with multicollinearity and for performing variable selection.
- **Partial Least Squares (PLS)** regression projects both predictors and response variables to a latent space, maximizing their covariance. It is especially suitable for spectral data due to its ability to handle high-dimensional and collinear variables.
- **Support Vector Regression (SVR)** models non-linear relationships by transforming data into a higher-dimensional space using kernel functions. It aims to fit the best hyperplane within a tolerance margin.
- **LassoLarsCV** uses the lasso and the Least Angle Regression (LARS) algorithms with built-in cross-validation to select the optimal amount of L1 regularization. It encourages sparsity and aids in automatic feature selection.
- **RidgeCV** applies L2 regularization and selects the best regularization parameter using cross-validation. It is robust against multicollinearity and can stabilize coefficient estimates.
- **Gradient Boosting Machines (GBMs)** is a powerful ensemble method that builds a sequence of weak learners, typically decision trees, to minimize prediction error. It incrementally fits residuals from previous models to improve overall performance.

Each model was trained on the optimally preprocessed data and evaluated on test data. The hyperparameters of each model were tuned using Bayesian optimization to maximize predictive performance. To prevent data leakage, all steps including hyperparameter optimization were strictly confined to the training data.

### 2.6. Hyperparameter Tuning Strategy

We adopted a two-stage optimization framework that decouples preprocessing pipeline optimization from final model hyperparameter tuning to balance computational efficiency and modeling flexibility.

During the first stage, preprocessing pipelines were optimized using Bayesian optimization with cross-validation, where each pipeline configuration was evaluated using a RidgeCV regression model. RidgeCV was selected as the estimator during this stage due to its single hyperparameter and efficient internal cross-validation. This allowed the framework to explore a wide variety of preprocessing configurations without the added computational burden of simultaneously tuning complex model architectures. The θ values were searched over a log-spaced range from $10^{-6}$ to $10^{6}$. The values of $n_{init}$ and $n_{iter}$ were set to 50 and 200, respectively.

Table 1 summarizes the hyperparameter search space for a few of the preprocessing components explored during optimization.

In the second stage, the best performing preprocessing pipelines were fixed and used to evaluate multiple regression models. These included Partial Least Squares (PLS), Elastic Net, RidgeCV, LassoLarsCV, Support Vector Regression (SVR), and Gradient Boosting Machines (GBMs). Each model except RidgeCV and LassoLarsCV underwent hyperparameter tuning using Bayesian optimization on the training data, with the search spaces listed in Table 2. The values of $n_{init}$ and $n_{iter}$ were set at 5 and 100, respectively, for all models, except PLS, where they were set to 5 and 10.

This two-stage procedure provides a clear separation between preprocessing pipeline discovery and model learning, enabling flexible experimentation while keeping overall search complexity tractable. Moreover, models like Elastic Net and LassoLarsCV inherently perform feature selection by assigning zero weights to less informative variables, offering indirect insights into variable importance.

### 2.7. Statistical Analysis

To evaluate whether the optimized preprocessing pipeline statistically outperformed baseline methods, we conducted hypothesis testing on fold-level RMSE values from 5-fold GroupShuffleSplit cross-validation across models, and milk components was conducted. We restricted the analysis to the PLS and RidgeCV models, as they yielded the best overall results. This led to a total of 24 pairwise comparisons (2 models × 4 components × 3 comparisons).

For each comparison, we tested the following hypotheses:

$$\begin{array}{cc} H_{0} & :\mu_{\mathrm{optimized}}-\mu_{\mathrm{baseline}}=0\;(\mathrm{no}\;\mathrm{difference}\;\mathrm{in}\;\mathrm{mean}\;\mathrm{RMSE})\\ H_{1} & :\mu_{\mathrm{optimized}}-\mu_{\mathrm{baseline}}\neq 0\;(\mathrm{significant}\;\mathrm{difference}\;\mathrm{in}\;\mathrm{mean}\;\mathrm{RMSE}) \end{array}$$

The choice of statistical test was based on the normality of the paired RMSE differences, assessed using the Shapiro–Wilk test (*p* > 0.05). If normality held, we used a paired t-test; otherwise, the Wilcoxon signed-rank test was applied [43,44].

To control the family-wise error rate from multiple comparisons, we applied the Bonferroni correction to the resulting *p*-values [45]. Cohen’s d was computed to quantify the effect size and direction of each comparison, with negative values indicating better performance (lower RMSE) by the optimized pipeline. The absolute magnitude of *d* follows conventional benchmarks: values greater than 0.8 denote a large effect size, and values exceeding 1.3 are considered very large [46]. We also reported 95% confidence intervals for the mean RMSE differences. All tests were two-sided, with a corrected significance threshold of α=0.05.

Boxplots showing RMSE distributions for each preprocessing method and milk component were generated to visually support the statistical findings, with asterisks indicating significance levels (* p<0.05, ** p<0.01 after Bonferroni correction).

## 3. Results and Discussion

### 3.1. Dataset Statistics and Distribution

Table 3 provides summary statistics for the four milk components analyzed: fat, protein, lactose, and total solids. Among these, total solids show the highest mean concentration (16.15%), followed by fat (5.57%), protein (4.76%), and lactose (4.56%). Protein and lactose display narrow standard deviations (0.51 and 0.12, respectively), indicating relatively consistent composition across samples, whereas fat and total solids show more variability. The lower bounds for fat and total solids (3.07% and 12.11%, respectively) also suggest possible sample dilution or formulation effects.

**Figure 2:**
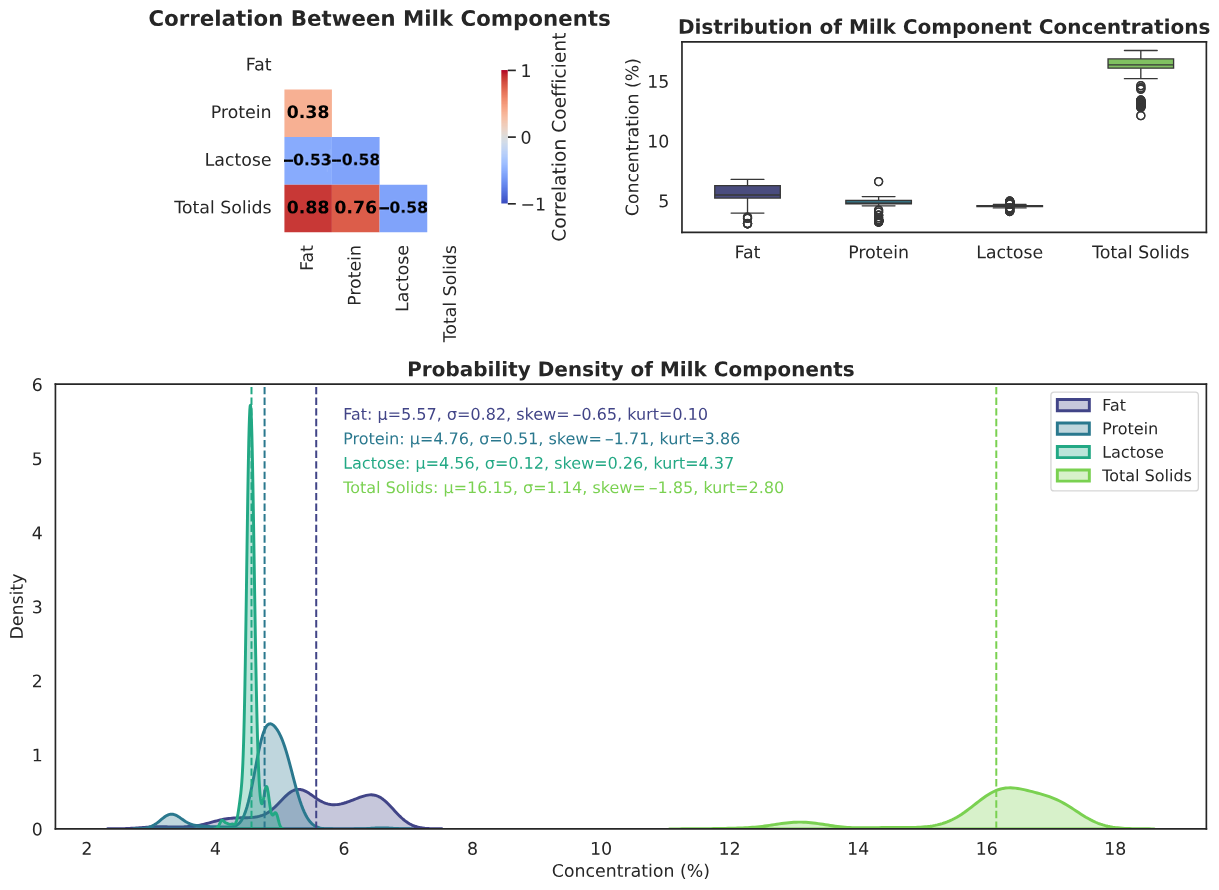
Distribution of milk components with their statistical properties.

Figure 2 provides a visual overview of the relationships and distribution patterns among the analyzed milk components (fat, protein, lactose, and TS). The correlation matrix (top left) reveals a strong positive correlation between fat and total solids (r=0.88), indicating that higher fat content tends to be associated with higher total solids, an expected trend in milk composition. A moderate negative correlation is observed between protein and lactose (r=−0.58), suggesting that as protein levels increase, lactose concentrations may slightly decrease. Additional correlations include a moderate positive relationship between protein and total solids (r=0.76), and a weak positive correlation between fat and protein (r=0.38).

The boxplots (top right) confirm that protein and lactose concentrations are relatively uniform across samples, whereas fat and total solids show broader variability which may reflect processing practices or targeted composition adjustment in the sample set.

Lastly, the density plots (bottom) show that most components are negatively skewed, particularly protein and total solids, suggesting the majority of samples cluster near upper concentration ranges. Elevated kurtosis values (e.g., 3.86 for protein and 4.37 for lactose) indicate peaked distributions with a few low-value outliers. These trends point toward controlled or processed milk samples rather than fresh raw milk, which is often subject to greater component variation.

### 3.2. Spectral Preprocessing

To mitigate the risk of overfitting and enhance model robustness, we explored spectral preprocessing pipelines with a restricted number of steps, using our automated optimization framework based on Bayesian optimization. Two configurations were considered by setting the allowed preprocessing pipeline length to either [1,2] (allows for either single preprocessing steps or combinations of two compatible methods) or [1,2,3] (allows for either single preprocessing steps or combinations of two or three compatible methods).

The optimization was performed solely on the training set, leveraging group-aware cross-validation using the Group-Shuffle-Split method to respect sample dependencies. We employed Bayesian optimization with $n_{\mathrm{init}}=50$ and $n_{\mathrm{iter}}=200$. All preprocessing steps were drawn from the following set: MSC, SavGol, detrend, scaler, SNV, robust_scaler, EMSC, PCA, normalization, autoscale, globalscaler, and meancn.

The optimized preprocessing pipelines for each milk component are summarized in Table 4 for the [1,2] and [1,2,3] configurations, respectively. Interestingly, the optimal pipelines were identical across both configurations, suggesting that a simpler preprocessing structure was sufficient for our data.

The consistency of results across both experimental configurations supports the robustness of the identified preprocessing schemes.

**Figure 3:**
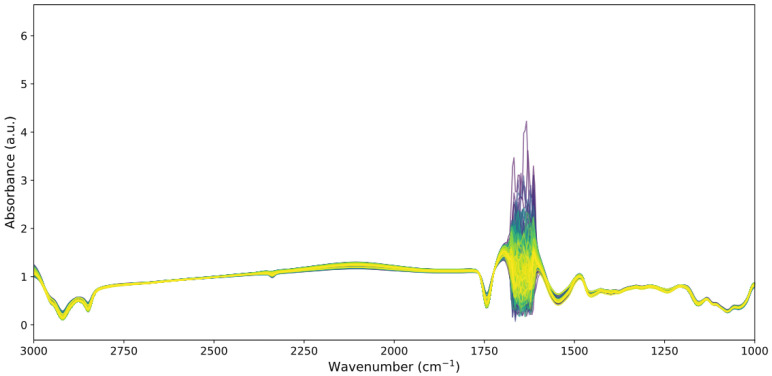
Raw MIR spectra of 135 calibration milk samples in the 3000–1000 cm^−1^ region.

**Figure 4:**
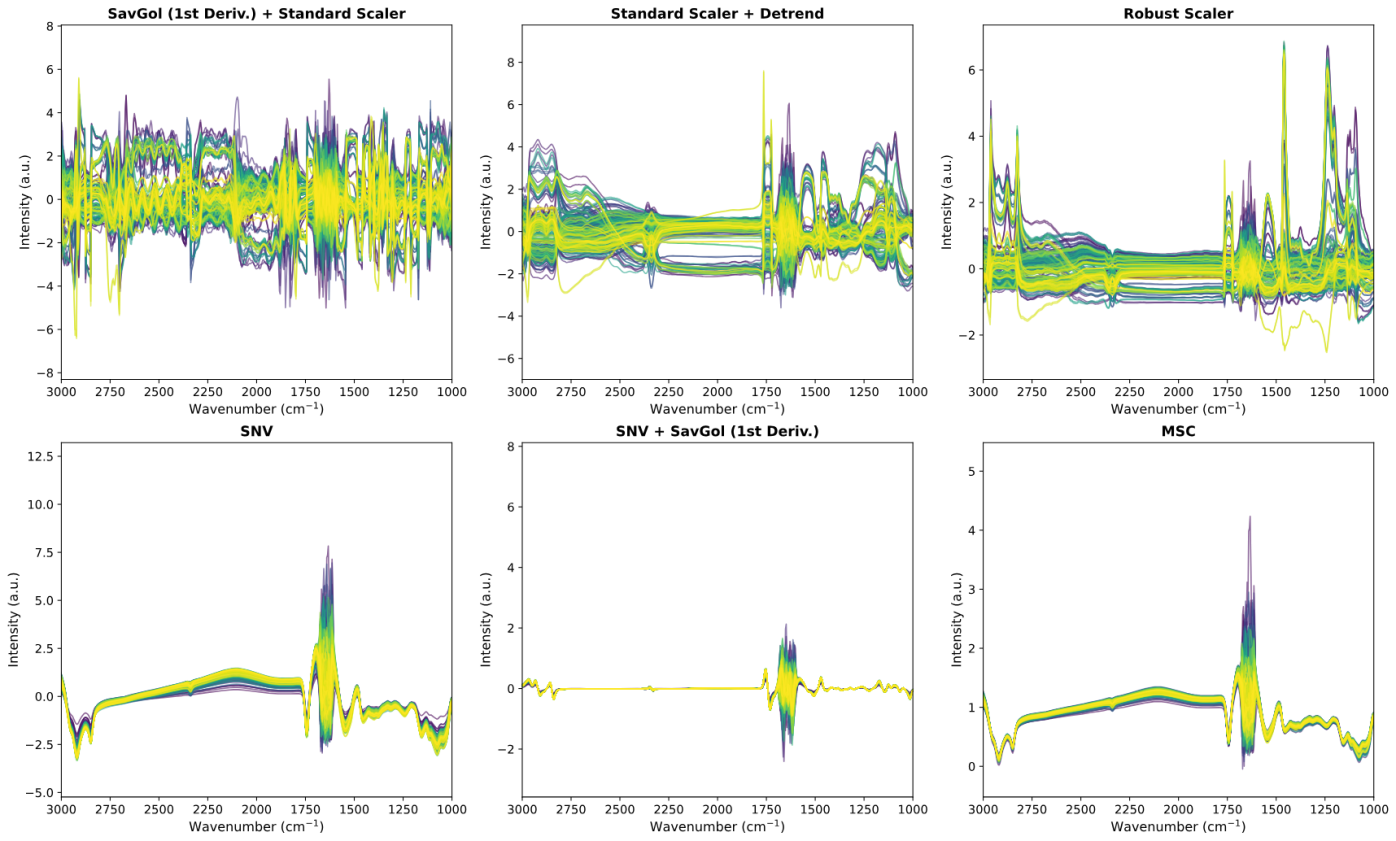
Comparison of spectral preprocessing pipelines. Top: automated, data-driven pipelines identified via Bayesian optimization. Bottom: commonly used literature-based methods (e.g., SNV, MSC, and derivatives) [25,30,31,47,48].

Figure 3 and  Figure 4 provide a visual comparison of the raw spectral data and the effects of various preprocessing strategies.

Figure 3 presents the raw MIR spectra of 135 milk calibration samples, which exhibit high overall alignment and minimal baseline or scatter artifacts. The spectra are smooth and consistent across the 3000–1000 cm^−1^ range, with major absorbance bands clearly preserved. A localized region of high-frequency variation is visible between approximately 1750 cm^−1^ and 1600 cm^−1^, likely reflecting chemical variability or instrument-related noise in that spectral window.

In Figure 4, the top row displays preprocessing pipelines optimized via Bayesian optimization and tailored for individual milk components, while the bottom row includes commonly reported literature methods such as SNV, MSC, and their combinations with derivatives. Literature-based techniques (e.g., SNV and MSC) effectively smooth the spectra and suppress global variation, resulting in visually cleaner profiles. However, this visual uniformity can come at the cost of reducing predictive information, particularly if relevant spectral variability is filtered out.

Conversely, the optimized pipelines introduced sharper variations especially in regions like 1450–1250 cm^−1^ and 2250–1750 cm^−1^ due to the application of scalers and derivatives. While these transformations may appear noisier, they are selected based on their ability to enhance model-relevant features rather than aesthetic smoothness. This contrast reinforces the core philosophy behind the Bayesian optimization approach: preprocessing should be optimized for predictive performance, not visual clarity.

### 3.3. Regression Analysis and the Importance of Optimized Preprocessing

To assess the impact of preprocessing techniques on predicting milk component concentrations on the test set, we conducted regression analyses under three distinct scenarios: without preprocessing, with optimized preprocessing obtained via Bayesian optimization, and using the custom preprocessing techniques previously reported in the literature (MSC, SNV, first derivative, and second derivative).

From Table 5, without preprocessing (baseline scenario), predictive models yielded reasonably accurate results on the test set. For example, fat prediction achieved an RMSEP of 0.159 (PLS regression, $R^{2}=0.903$), protein showed high prediction accuracy with an RMSEP of 0.063 (LassoLarsCV, $R^{2}=0.974$), lactose presented a moderate predictive accuracy (RMSEP = 0.027, PLS regression, $R^{2}=0.912$), and total solids predictions demonstrated robust accuracy (RMSEP = 0.158, PLS regression, $R^{2}=0.958$).

Applying optimized preprocessing improved model performance for protein and lactose predictions. Specifically, protein prediction RMSEP decreased to 0.054 (RidgeCV regression, $R^{2}=0.981$), enhancing predictive accuracy compared to the baseline scenario. Similarly, lactose prediction benefited from preprocessing optimization, achieving a lower RMSEP of 0.026 (PLS regression, $R^{2}=0.917$). Total solids and fat predictions also showed moderate improvements, with the best total solids prediction yielding an RMSEP of 0.154 (RidgeCV regression, $R^{2}=0.960$) and fat prediction reaching an RMSEP of 0.139 (RidgeCV regression, $R^{2}=0.926$).

Notably, across all three scenarios, support vector regression (SVR) consistently underperformed on the test set, despite often achieving strong performance on the training data. This discrepancy highlights the risk of overfitting when using highly flexible models on relatively limited datasets. The use of group-aware cross-validation during model development proved effective in providing a more realistic assessment of model generalization ability, particularly where reserving a separate internal validation set was not practical.

**Figure 5:**
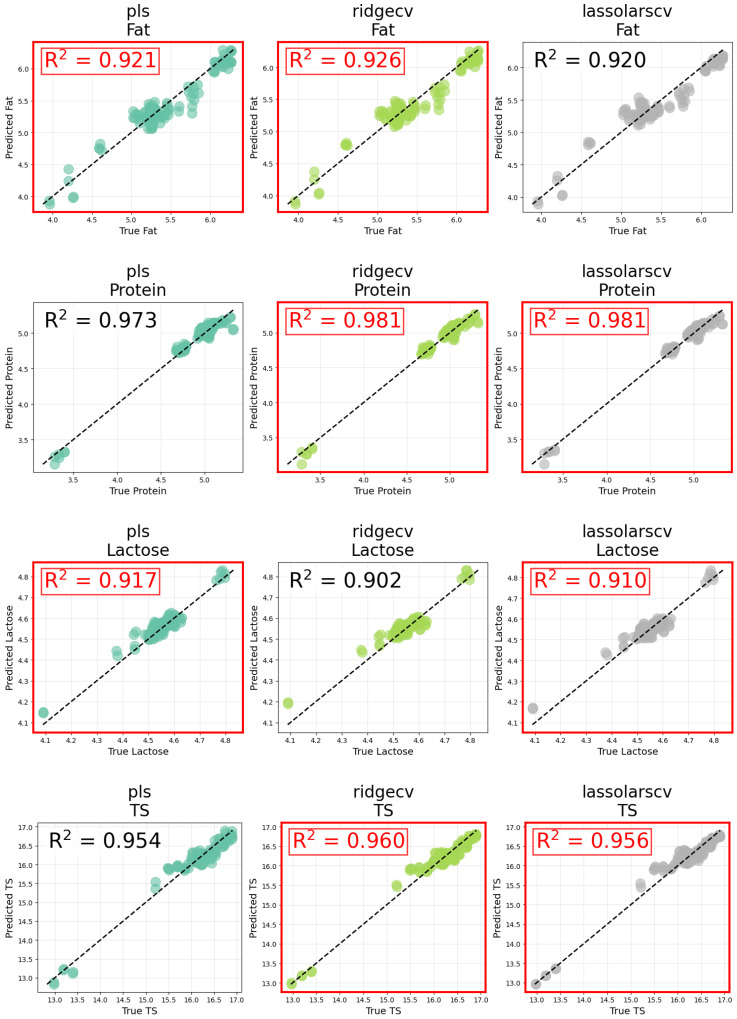
Predicted vs. true value scatter plots for each milk component using optimized preprocessing obtained via Bayesian optimization. This figure includes the results for the top-performing models (PLS, RidgeCV, and LassoLarsCV). Models with the highest $R^{2}$ for each component are outlined in red.

**Figure A1:**
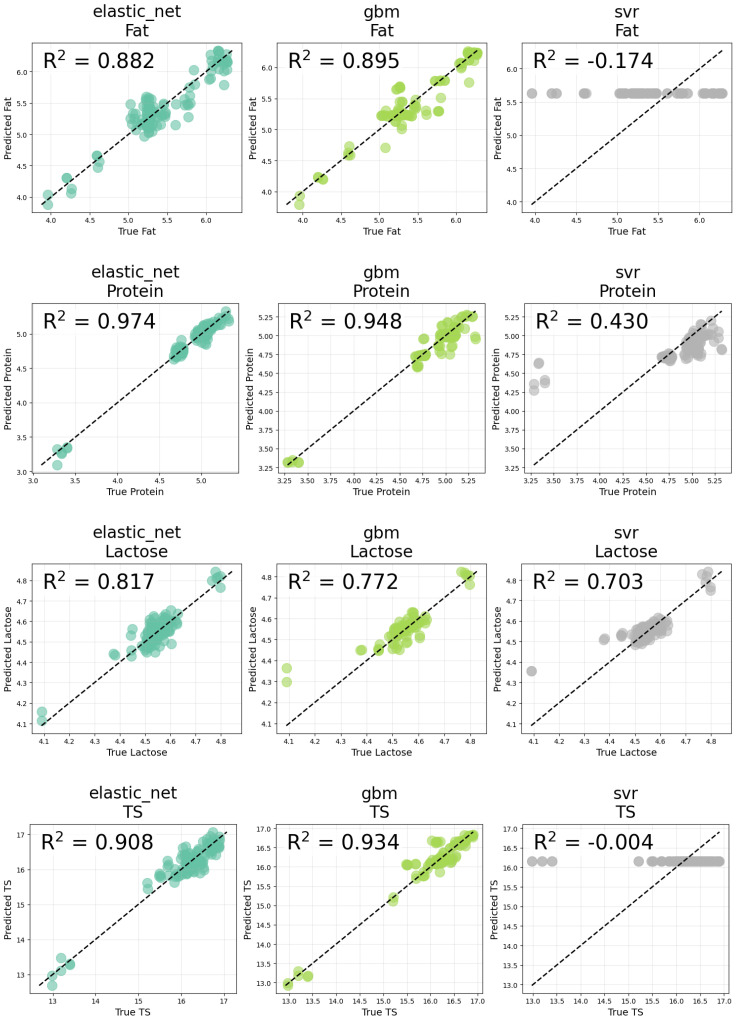
Predicted vs. true value scatter plots for each milk component using optimized preprocessing obtained via Bayesian optimization. This figure presents results for additional models: SVR, GBM, and ElasticNet.

These findings are visually confirmed in Figure 5, which displays predicted versus true plots for three representative models, PLS, RidgeCV, and LassoLarsCV on the test set using optimized preprocessing obtained through Bayesian optimization. The best-performing models (highlighted in red) align closely with the identity line, especially for protein and total solids. Results for the remaining models i.e SVR, GBM, and ElasticNet, are included in Appendix C (Figure A1). The full regression performance metrics on both the training and test sets for each model and preprocessing strategy are provided in Appendix B (Table A1, Table A2 and Table A3).

From Table 6, using custom preprocessing methods commonly reported in the literature, we identified several studies that applied spectroscopy techniques to milk datasets. Zhu et al. [25] and Wu et al. [47] both reported SNV as the optimal preprocessing technique for their respective datasets. Wu et al. specifically employed short-wave NIR spectroscopy in the 800–1050 nm range to analyze the primary compounds in milk powder. Similarly, Amsaraj et al. [30] and Bonfatti et al. [48] identified a combination of SNV and first-derivative Savitzky–Golay (SavGol) filtering as their optimal preprocessing approach. Bonfatti et al. [48] specified SavGol parameters as a window length of 15, derivative order of 1, and polynomial order of 4. As Amsaraj et al. [30] did not report their SavGol parameters, we adopted the same values for consistency.

Although the literature has suggested that MSC and SNV are generally effective in improving model performance, our results contradict this assumption On our dataset, these methods produced inferior outcomes compared to both unprocessed data and the results achieved through our optimized preprocessing pipeline. Similarly, the use of first and second derivatives, often recommended for enhancing predictive power, offered only marginal benefits over no preprocessing in certain cases The best fat prediction using custom preprocessing (SNV + 1st Der SavGol, PLS, $R^{2}=0.896$), as used by Amsaraj et al. [30] and Bonfatti et al. [48], was lower than both no preprocessing (PLS, $R^{2}=0.903$) and our method (RidgeCV, $R^{2}=0.926$). For protein, the top custom result (MSC, RidgeCV, $R^{2}=0.972$) from Inon et al. [31] also underperformed compared to no preprocessing (LassoLarsCV, $R^{2}=0.974$) and our method (RidgeCV, $R^{2}=0.981$). Lactose prediction using SNV + 1st Der SavGol (LassoLarsCV, $R^{2}=0.846$) similarly lagged behind no preprocessing (PLS, $R^{2}=0.912$) and our approach (PLS, $R^{2}=0.917$). For total solids, MSC with LassoLarsCV ($R^{2}=0.950$) was outperformed by both no preprocessing (PLS, $R^{2}=0.958$) and our method (RidgeCV, $R^{2}=0.960$).

These comparative findings underscore the critical importance of dataset-specific preprocessing optimization. Adopting preprocessing methods from unrelated or even closely related prior studies without validation can negatively affect prediction accuracy. Thus, optimized preprocessing tailored explicitly to individual datasets and prediction targets remain an essential step for achieving maximum accuracy and reliability in milk component prediction models.

### 3.4. Statistical Comparison of Preprocessing Methods

Statistical testing confirmed that the optimized pipeline significantly outperformed baseline preprocessing methods in most milk components as presented in Table 7. Before Bonferroni’s correction, almost all pairwise comparisons were statistically significant at the level α=0.05; this included all comparisons under RidgeCV and 8 of 12 under PLS. The normality of RMSE differences was assessed using the Shapiro–Wilk test for all pairwise comparisons. A paired t-test was used in all cases except one under PLS, where normality was violated; in that instance, the nonparametric Wilcoxon signed-rank test was applied.

After applying the Bonferroni correction, eight of nine RidgeCV comparisons remained significant, with the exception of Total Solids. In particular, three comparisons exhibited strong significance at the p<0.01 level: fat (optimized vs. SNV+SG) (p=0.0047), and true protein (optimized vs. MSC) (p=0.0023) and (optimized vs. SNV) (p=0.0062). In contrast, only one PLS comparison remained significant after correction, despite strong trends observed prior.

All RidgeCV comparisons produced large to extremely large effect sizes (Cohen’s |d| ranging from 2.3 to 7.4), supporting the practical relevance of the optimized pipeline. PLS comparisons also consistently showed large effect sizes despite losing corrected significance.

**Figure 6:**
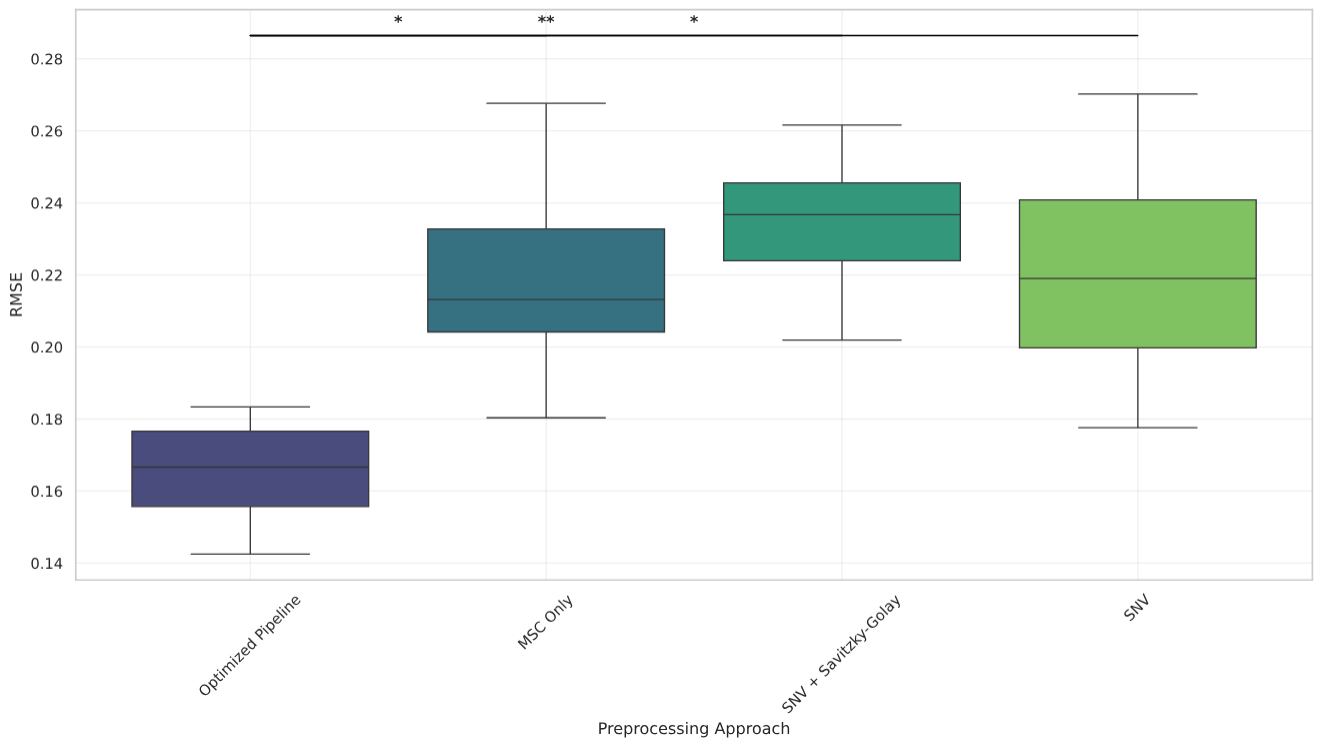
RMSE distribution across preprocessing methods for the fat (%) component. The optimized pipeline achieved the lowest median RMSE. Statistical significance is annotated using * *p* < 0.05 and ** *p* < 0.01 (Bonferroni-corrected).

**Figure 7:**
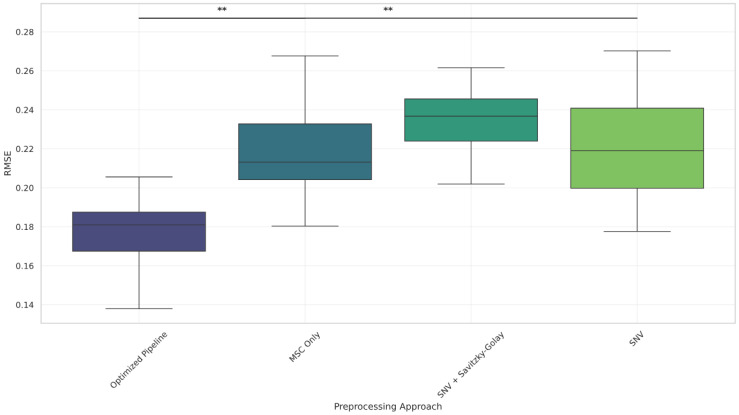
RMSE distribution across preprocessing methods for the true protein (%) component. Significant differences favor the optimized pipeline. Significance levels are marked as and ** *p* < 0.01 (Bonferroni-corrected).

**Figure 8:**
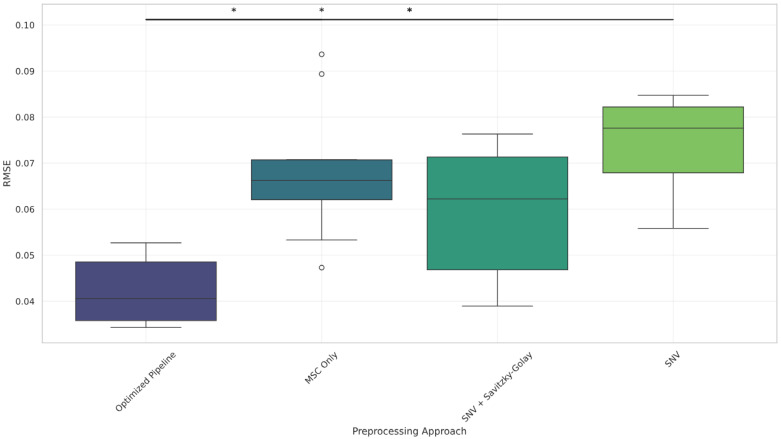
RMSE distribution across preprocessing methods for the Lactose (%) component. Optimized Pipeline outperforms baselines with statistically significant differences. * *p* < 0.05, (Bonferroni-corrected).

**Figure A2:**
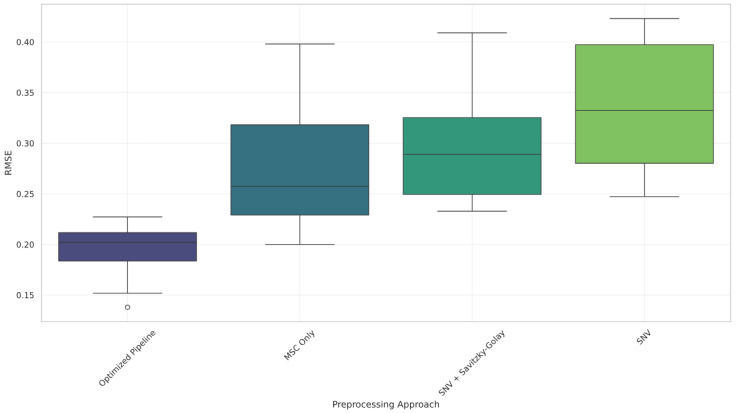
RMSE distribution across preprocessing methods for the total solids (TS %) component. The optimized pipeline shows the most consistent and lowest RMSE values. (Bonferroni-corrected).

Boxplots (Figure 6, Figure 7 and Figure 8 and Figure A2) further illustrate these results, showing consistent reductions in RMSE and variability for the optimized pipeline across components. Even for total solids, where Bonferroni corrected significance was not observed, the optimized pipeline exhibited a visibly lower RMSE distribution compared to those for all other methods.

This study highlights the pivotal role of spectral preprocessing in improving the accuracy of milk component predictions. Using a Bayesian optimization-based framework, we identified preprocessing pipelines that consistently outperformed both no preprocessing and alternative algorithms reported in the literature, especially for protein and lactose. Fat and total solids have stronger IR spectral signatures, and we observed more modest gains, suggesting that these analytes may require simpler corrections.

A major insight is the data- and component-specific nature of preprocessing. Optimal pipelines vary between components, confirming that a universal approach is inadequate. This aligns with previous work in spectroscopy, such as Vestergaard et al. [49], which found that no single preprocessing strategy was the best across analytes. Our findings further show that commonly used methods (e.g., MSC and SNV) underperform when applied without dataset-specific tuning, reinforcing the need for empirical evaluation.

The Bayesian optimization approach offers a significant advantage by automating preprocessing selection, reducing reliance on trial and error. This method efficiently explores the pipeline space, often identifying simple but highly effective two-step combinations that enhance both interpretability and generalizability. Moreover, the transparent and reproducible nature of this approach makes it suitable for broader spectroscopic applications beyond milk, including food quality control and authenticity testing.

## 4. Conclusions

We present a Bayesian optimization-based framework for selecting spectral preprocessing pipelines, demonstrating its utility in accurately determining the amounts of fat, protein, lactose, and total solids from MIR spectra of milk. By comparing optimized preprocessing to both unprocessed data and methods commonly reported in the literature, the Bayesian optimization approach consistently delivered improved regression accuracy, especially for protein and lactose. These findings underscore the benefit of data-driven preprocessing over heuristic or borrowed approaches.

A key strength of this framework lies in its ability to identify simple yet effective pipelines typically involving just two to three steps, eliminating the need for exhaustive manual tuning. This approach makes it especially valuable when seeking high performance without sacrificing interpretability or implementation simplicity. The immediate applicability of the method to MIR milk spectra suggests that even small accuracy gains can impact quality control and formulation relevant in industry settings.

Looking ahead, while the current analysis focused primarily on spectral preprocessing optimization, future work may involve incorporating wavenumber selection techniques as an additional step to further enhance predictive performance. Expanding the framework to support broader spectral domains and integrating it with model hyperparameter tuning could also promote the development of fully automated modeling pipelines. In summary, this approach provides a scalable framework for spectral data preparation, supporting robust and generalizable modeling across diverse chemometric applications.

## Figures and Tables

**Table 1:** Hyperparameter search space for preprocessing components used during optimization.

Preprocessing	Parameter	Search Space	Description
RobustScaler	quantile_range	[5, 80], [10, 90], [25, 75]	Interquantile scaling range
	with_centering	True, False	Whether to center data before scaling
	with_scaling	True, False	Whether to scale data
	unit_variance	True, False	Whether to scale to unit variance
StandardScaler	with_mean	True, False	Centering option
	with_std	True, False	Scaling option
SavitzkyGolay	window_length	5, 7, 11, 15	Smoothing window size
	polyorder	2, 3, 4	Polynomial order
MultiplicativeScatterCorrection	reference	mean, median	Reference spectrum type
Detrend	method	simple, polynomial, spline	Detrending method
	order	integer	The order of the polynomial or spline fit

**Table 2:** Hyperparameter search space for regression models used in the final evaluation.

Model	Parameter	Search Space	Description
PLS	n_components	2 to min(10,n_features)	Number of latent variables
SVR	C	0.1–100	Regularization strength
	epsilon	0.01–1.0	Epsilon-tube width
	gamma	$10^{-4}$–1.0 (log-scale)	Kernel coefficient for RBF
RidgeCV	θ	$10^{-6}$–$10^{6}$ (log-scale)	Regularization values
LassoLarsCV	cv	5 (fixed)	Number of CV folds
Elastic Net	l1_ratio	0.1–0.9	Balance of L1 vs. L2 penalty
	alpha	$10^{-4}$–1.0	Regularization strength
GBM	n_estimators	50–300	Number of boosting rounds
	learning_rate	0.01–0.3	Step size shrinkage
	max_depth	3–10	Maximum tree depth
	min_samples_split	2–10	Minimum samples to split node

**Table 3:** Descriptive statistics for milk components: fat, protein, lactose, and total solids.

Metric	Fat	Protein	Lactose	Total Solids
Count	385	385	385	385
Mean	5.5679	4.7644	4.5607	16.1490
Std	0.8178	0.5065	0.1152	1.1442
Min	3.0700	3.1900	4.0900	12.1100
25%	5.2167	4.7400	4.5100	16.0800
50%	5.4750	4.7900	4.5500	16.3560
75%	6.2625	5.0225	4.5900	16.8500
Max	6.7750	6.5960	4.9517	17.5600

**Table 4:** Optimal preprocessing pipelines identified through automated Bayesian optimization using allowed combinations [1,2] and [1,2,3].

Component	Preprocessing Step(s)	Parameters
Fat	savgol → scaler	deriv_order = 1, filter_win = 13,
		poly_order = 5, with_mean = True,
		with_std = True
Lactose	scaler → detrend	with_mean = True, with_std = True
		method = “polynomial”, order = 2,
		dspline = 100
Protein	robust_scaler	quantile_range = [5, 80],
		with_scaling = True, unit_variance = False
TS	savgol → scaler	deriv_order = 1, filter_win = 13,
		poly_order = 5 with_mean = True,
		with_std = True

**Table 5:** Regression performance comparison between no preprocessing and optimized preprocessing obtained via Bayesian optimization.

	No Preprocessing				Optimized Preprocessing			
Component	Best Model	RMSEP	$R^{2}$	Method	Best Model	RMSEP	$R^{2}$	Method
Fat	PLS	0.159	0.903	None	RidgeCV	0.139	0.926	Optimized
Protein	LassoLarsCV	0.063	0.974	None	RidgeCV	0.054	0.981	Optimized
Lactose	PLS	0.027	0.912	None	PLS	0.026	0.917	Optimized
Total Solids	PLS	0.158	0.958	None	RidgeCV	0.154	0.960	Optimized

**Table 6:** Comparison of best literature-based preprocessing methods across milk components.

	Zhu et al. [25], Wu et al. [47]		Amsaraj et al. [30], Bonfatti et al. [48]		Inon et al. [31]	
Component	Best Method	$R^{2}$	Best Method	$R^{2}$	Best Method	$R^{2}$
Fat	SNV (PLS)	0.882	SNV + 1st Der SavGol (PLS)	0.896	MSC (PLS)	0.891
Protein	SNV (Elastic Net)	0.960	SNV + 1st Der SavGol (LassoLarsCV)	0.970	MSC (RidgeCV)	0.972
Lactose	SNV (Elastic Net)	0.835	SNV + 1st Der SavGol (LassoLarsCV)	0.846	MSC (RidgeCV)	0.822
Total Solids	SNV (RidgeCV)	0.917	SNV + 1st Der SavGol (PLS)	0.933	MSC (LassoLarsCV)	0.950

**Table 7:** Statistical comparison of the optimized pipeline with baseline preprocessing methods. Significance is indicated for both uncorrected and Bonferroni-corrected *p*-values.

Component	Comparison	Cohen’s D	P	Sig. (P)	P (Corr.)	Sig. (Corr.)
Fat	MSC Only	−3.43	0.001	Yes	0.017	Yes
Fat	SNV + SG	−7.43	0.000	Yes	0.005	Yes
Fat	SNV	−3.52	0.001	Yes	0.024	Yes
Lactose	MSC Only	−3.38	0.002	Yes	0.044	Yes
Lactose	SNV + SG	−4.47	0.001	Yes	0.025	Yes
Lactose	SNV	−4.99	0.001	Yes	0.027	Yes
TS	MSC Only	−3.37	0.011	Yes	0.266	No
TS	SNV + SG	−2.91	0.013	Yes	0.314	No
TS	SNV	−3.57	0.005	Yes	0.120	No
True Protein	MSC Only	−2.33	0.000	Yes	0.002	Yes
True Protein	SNV + SG	−4.04	0.003	Yes	0.070	No
True Protein	SNV	−2.41	0.000	Yes	0.006	Yes

## Data Availability

The milk spectral dataset used in this study was obtained from Agropur and is available upon reasonable request from the authors. The Bayesian optimization framework is open-source and can be installed via pip or conda. The source code and documentation are available at https://github.com/habeeb3579/Spectoprep (accessed on 4 May 2025).

## Appendix A

**Algorithm A1** Automated spectroscopic data pipeline optimization.
1: **Input:** Training data $X_{train}\in R^{n\times p}$, $y_{train}\in R^{n}$ 2: **Input:** Optional test data $X_{test}\in R^{m\times p}$, $y_{test}\in R^{m}$ 3: **Input:** Preprocessing steps $S=\{s_{1},\ldots ,s_{k}\}$, incompatibility sets $I=\{I_{1},\ldots ,I_{l}\}$ 4: **Input:** Maximum pipeline length $L_{max}$, allowed lengths $L\subseteq \{1,\ldots ,L_{max}\}$ 5: **Input:** Parameter bounds $\Theta =\{\theta_{1},\ldots ,\theta_{d}\}$ 6: **Input:** Bayesian optimization parameters: initial points $n_{init}$, iterations $n_{iter}$ 7: **procedure** GeneratePipelineConfigurations(S,I,L) 8: P←∅ 9: **for** l∈L **do** 10: $C_{l}\leftarrow$ all combinations of *l* elements from S 11: **for** pipeline $p\in C_{l}$ **do** 12: valid ← True 13: **for** $I_{j}\in I$ **do** 14: **if** $|p\cap I_{j}|>1$ **then** 15: valid ← False 16: **break** 17: **end if** 18: **end for** 19: **if** valid **then** 20: P←P∪{p} 21: **end if** 22: **end for** 23: **end for** 24: **return** P 25: **end procedure** 26: P←GeneratePipelineConfigurations(S,I,L) 27: $N_{p}\leftarrow \left|P\right|$ ▹ Number of valid pipelines 28: **procedure** BuildPipeline(i,θ) 29: p←P[i] 30: pipeline ← empty Pipeline 31: **for** $s_{j}\in p$ **do** 32: preprocessor_*j*_← BuildPreprocessor($s_{j}$, θ) 33: Add $\left(s_{j},{\mathrm{preprocessor}}_{j}\right)$ to pipeline 34: **end for** 35: Add (“ridge”, Ridge(α=θ[“ridge_alpha”])) to pipeline 36: **return** pipeline 37: **end procedure** 38: **procedure** EvaluateObjective(θ) 39: i←⌊θ[“pipeline_config”]+0.5⌋ 40: $i\leftarrow \max(0,\min\left(i,N_{p}-1\right))$ 41: pipeline ←BuildPipeline(i,θ) 42: **if** $X_{test}\neq \mathrm{None}$ **and** $y_{test}\neq \mathrm{None}$ **then** 43: Fit pipeline on $X_{train}$, $y_{train}$ 44: ${\hat{y}}_{test}\leftarrow \mathrm{pipeline}.\mathrm{predict}\left(X_{test}\right)$ 45: ${\mathrm{RMSE}}_{test}\leftarrow \sqrt{\frac{1}{m}\sum_{j=1}^{m}{\left(y_{test,j}-{\hat{y}}_{test,j}\right)}^{2}}$ 46: $R_{test}^{2}\leftarrow 1-\frac{\sum_{j=1}^{m}{\left(y_{test,j}-{\hat{y}}_{test,j}\right)}^{2}}{\sum_{j=1}^{m}{\left(y_{test,j}-{\bar{y}}_{test}\right)}^{2}}$ 47: score $\leftarrow -{\mathrm{RMSE}}_{test}$ 48: **else** 49: Initialize cross-validation splitter CV 50: predictions ←[], actuals ←[] 51: **for** (train_idx, val_idx) in CV.split($X_{train}$, $y_{train}$, groups) **do** 52: $X_{train}^{fold}\leftarrow X_{train}$[train_idx] 53: $y_{train}^{fold}\leftarrow y_{train}$[train_idx] 54: $X_{val}^{fold}\leftarrow X_{train}$[val_idx] 55: $y_{val}^{fold}\leftarrow y_{train}$[val_idx] 56: Fit pipeline on $X_{train}^{fold}$, $y_{train}^{fold}$ 57: ${\hat{y}}_{val}^{fold}\leftarrow \mathrm{pipeline}.\mathrm{predict}\left(X_{val}^{fold}\right)$ 58: Append ${\hat{y}}_{val}^{fold}$ to predictions 59: Append $y_{val}^{fold}$ to actuals 60: **end for** 61: **if** predictions is empty **then** 62: **return** $-10^{6}$ ▹ Penalty for failure 63: **end if** 64: ${\mathrm{RMSE}}_{val}\leftarrow \sqrt{\frac{1}{n}\sum_{i=1}^{n}{\left(y_{i}-{\hat{y}}_{i}\right)}^{2}}$ 65: score $\leftarrow -{\mathrm{RMSE}}_{val}$ 66: **end if** 67: **return** score 68: **end procedure** 69: **procedure** BayesianOptimize ($\mathrm{EvaluateObjective},\Theta ,n_{init},n_{iter}$) 70: Initialize Bayesian optimizer with bounds Θ 71: Evaluate $n_{init}$ random points 72: **for** i=1 to $n_{iter}$ **do** 73: Select next point $\theta^{\left(i\right)}$ 74: Evaluate $\mathrm{EvaluateObjective}(\theta^{\left(i\right)})$ 75: Update surrogate model 76: **end for** 77: $\theta^{*}\leftarrow$ best parameters found 78: $i^{*}\leftarrow \left\lfloor \theta^{*}\left[“\mathrm{pipeline\_config}”\right]+0.5\right\rfloor$ 79: $p^{*}\leftarrow P\left[i^{*}\right]$ 80: best_pipeline $\leftarrow \mathrm{BuildPipeline}(i^{*},\theta^{*})$ 81: Fit best_pipeline on full $X_{train}$, $y_{train}$ 82: **return** best_pipeline, $\theta^{*}$ 83: **end procedure** 84: best_pipeline, best_params ←BayesianOptimize(EvaluateObjective, Θ, $n_{init}$, $n_{iter}$) 85: **if** $X_{test}\neq$ None **then** 86: ${\hat{y}}_{test}\leftarrow \mathrm{best\_pipeline.predict}\left(X_{test}\right)$ 87: ${\mathrm{RMSE}}_{test}\leftarrow \sqrt{\frac{1}{m}\sum_{i=1}^{m}{\left(y_{test,i}-{\hat{y}}_{test,i}\right)}^{2}}$ 88: $R_{test}^{2}\leftarrow 1-\frac{\sum_{i=1}^{m}{\left(y_{test,i}-{\hat{y}}_{test,i}\right)}^{2}}{\sum_{i=1}^{m}{\left(y_{test,i}-{\bar{y}}_{test}\right)}^{2}}$ 89: **end if** 90: **Output:** Best pipeline, best parameters, performance metrics

## Appendix B

**Table A1:** Full regression performance on both training and test sets for each milk component after Bayesian optimization-based preprocessing. Metrics include $R^{2}$ and RMSE for each model evaluated.

Component	Model	$R_{\mathrm{train}}^{2}$	RMSE_train_	$R^{2}P$	RMSEP
Fat	lassolarscv	0.9883	0.0826	0.9200	0.1445
	ridgecv	0.9937	0.0733	0.9255	0.1394
	gbm	1.0000	$1.48\times 10^{-8}$	0.8950	0.1655
	pls	0.9939	0.0712	0.9209	0.1437
	svr	0.9999	0.0011	−0.1742	0.5554
	elastic_net	0.9999	0.0024	0.8823	0.1753
Protein	lassolarscv	0.9966	0.0314	0.9807	0.0545
	ridgecv	0.9974	0.0277	0.9812	0.0539
	gbm	1.0000	$1.45\times 10^{-8}$	0.9483	0.0894
	pls	0.9893	0.0574	0.9728	0.0650
	svr	0.9994	0.0137	0.4304	0.2967
	elastic_net	0.9993	0.0015	0.9742	0.0631
Lactose	lassolarscv	0.9514	0.0273	0.9097	0.0275
	ridgecv	0.9492	0.0279	0.9018	0.0286
	gbm	1.0000	$1.48\times 10^{-8}$	0.7717	0.0437
	pls	0.9567	0.0257	0.9173	0.0263
	svr	0.9926	0.0011	0.7026	0.0499
	elastic_net	0.9972	0.0065	0.8173	0.0391
Total Solids (TS)	lassolarscv	0.9899	0.1274	0.9560	0.1615
	ridgecv	0.9906	0.1233	0.9602	0.1536
	gbm	1.0000	$1.47\times 10^{-8}$	0.9344	0.1973
	pls	0.9952	0.0879	0.9536	0.1659
	svr	0.9999	0.0119	−0.0041	0.7772
	elastic_net	0.9999	0.0021	0.9077	0.2340

**Table A2:** Full regression performance on both training and test sets for each milk component using no preprocessing.

Component	Model	$R_{\mathrm{train}}^{2}$	RMSE_train_	$R^{2}P$	RMSEP
Fat	lassolarscv	0.9687	0.0161	0.8622	0.1897
	ridgecv	0.9817	0.0123	0.8745	0.1810
	gbm	1.0000	0.0000	0.8792	0.1776
	pls	0.9846	0.0113	0.9033	0.1589
	svr	0.9999	0.0107	0.4262	0.3869
	elastic_net	0.9897	0.0093	0.8984	0.1628
Protein	lassolarscv	0.9978	0.0255	0.9743	0.0600
	ridgecv	0.9981	0.0236	0.9720	0.0657
	gbm	1.0000	0.0000	0.9483	0.0894
	pls	0.9893	0.0557	0.9727	0.0650
	svr	0.9994	0.0135	0.8354	0.1595
	elastic_net	0.9913	0.0503	0.9601	0.0786
Lactose	lassolarscv	0.9494	0.0278	0.7985	0.0410
	ridgecv	0.9894	0.0127	0.8233	0.0384
	gbm	1.0000	0.0000	0.8028	0.0451
	pls	0.9498	0.0277	0.9121	0.0271
	svr	0.9915	0.0114	0.3185	0.0755
	elastic_net	0.9562	0.0259	0.7620	0.0446
Total Solids (TS)	lassolarscv	0.9869	0.0145	0.9555	0.1625
	ridgecv	0.9885	0.0136	0.9537	0.1667
	gbm	1.0000	0.0000	0.9319	0.2010
	pls	0.9910	0.0120	0.9578	0.1582
	svr	0.9999	0.0118	0.8875	0.2584
	elastic_net	0.9934	0.0103	0.9493	0.1733

**Table A3:** Full regression performance on both training and test sets for each milk component using SNV preprocessing.

Component	Model	$R_{\mathrm{train}}^{2}$	RMSE_train_	$R^{2}P$	RMSEP
Fat	lassolarscv	0.9766	0.1393	0.8589	0.1919
	ridgecv	0.9784	0.1339	0.8472	0.1997
	gbm	1.0000	$1.46\times 10^{-8}$	0.7247	0.2680
	pls	0.9794	0.1305	0.8825	0.1751
	svr	0.9999	0.0011	−0.1742	0.5536
	elastic_net	0.9930	0.0760	0.8605	0.1908
Protein	lassolarscv	0.9597	0.1083	0.9565	0.0820
	ridgecv	0.9960	0.0341	0.9598	0.0789
	gbm	1.0000	$1.48\times 10^{-8}$	0.8915	0.1295
	pls	0.9757	0.0842	0.9490	0.0888
	svr	0.9994	0.0137	−0.2033	0.4312
	elastic_net	0.9929	0.0452	0.9597	0.0789
Lactose	lassolarscv	0.6510	0.7309	0.4230	0.0694
	ridgecv	0.9886	0.0132	0.8039	0.0404
	gbm	1.0000	$1.47\times 10^{-8}$	0.3484	0.0738
	pls	0.8741	0.4389	0.6422	0.0547
	svr	0.9916	0.0114	0.2172	0.0809
	elastic_net	0.9764	0.0190	0.8343	0.0372
Total Solids (TS)	lassolarscv	0.8836	0.4332	0.8491	0.2992
	ridgecv	0.9875	0.1418	0.9167	0.2222
	gbm	1.0000	$1.48\times 10^{-8}$	0.8831	0.2634
	pls	0.9796	0.1820	0.9167	0.2230
	svr	0.9999	0.0119	−0.0040	0.7772
	elastic_net	0.9994	0.0976	0.8988	0.2451

## Appendix C

**Table A4:** Complete statistical test results comparing the optimized pipeline with baseline preprocessing methods across all components and models. Includes effect sizes, confidence intervals, and both uncorrected and Bonferroni-corrected significance.

Model	Component	Comparison	Test	Statistic	P	Mean Diff	Cohen’s D	CI Lower	CI Upper	Sig. (P)	P (Corr.)	Sig. (Corr.)
pls	Fat	MSC Only	paired *t*-test	−4.30	0.013	−0.031	−2.61	−0.051	−0.011	Yes	0.304	No
pls	Fat	SNV + SG	paired *t*-test	−6.60	0.003	−0.047	−3.58	−0.066	−0.027	Yes	0.065	No
pls	Fat	SNV	paired *t*-test	−3.74	0.020	−0.032	−2.27	−0.056	−0.008	Yes	0.483	No
pls	Lactose	MSC Only	wilcoxon	0.00	0.063	−0.016	−2.06	−0.024	−0.008	No	1.000	No
pls	Lactose	SNV + SG	paired *t*-test	−2.13	0.100	−0.005	−0.70	−0.012	0.002	No	1.000	No
pls	Lactose	SNV	paired *t*-test	−7.16	0.002	−0.028	−3.01	−0.039	−0.017	Yes	0.048	Yes
pls	TS	MSC Only	paired *t*-test	−1.67	0.171	−0.017	−0.91	−0.045	0.011	No	1.000	No
pls	TS	SNV + SG	paired *t*-test	−4.11	0.015	−0.055	−3.22	−0.092	−0.018	Yes	0.353	No
pls	TS	SNV	paired *t*-test	−4.34	0.012	−0.091	−2.85	−0.150	−0.033	Yes	0.293	No
pls	True Protein	MSC Only	paired *t*-test	−6.15	0.004	−0.021	−1.23	−0.031	−0.012	Yes	0.085	No
pls	True Protein	SNV + SG	paired *t*-test	−2.76	0.051	−0.037	−2.02	−0.073	0.000	No	1.000	No
pls	True Protein	SNV	paired *t*-test	−4.90	0.008	−0.023	−1.18	−0.035	−0.010	Yes	0.193	No
ridgecv	Fat	MSC Only	paired *t*-test	−9.38	0.001	−0.077	−3.43	−0.099	−0.054	Yes	0.017	Yes
ridgecv	Fat	SNV + SG	paired *t*-test	−13.09	0.000	−0.091	−7.43	−0.111	−0.072	Yes	0.005	Yes
ridgecv	Fat	SNV	paired *t*-test	−8.61	0.001	−0.079	−3.52	−0.105	−0.054	Yes	0.024	Yes
ridgecv	Lactose	MSC Only	paired *t*-test	−7.33	0.002	−0.036	−3.38	−0.049	−0.022	Yes	0.044	Yes
ridgecv	Lactose	SNV + SG	paired *t*-test	−8.49	0.001	−0.029	−4.47	−0.038	−0.019	Yes	0.025	Yes
ridgecv	Lactose	SNV	paired *t*-test	−8.38	0.001	−0.036	−4.99	−0.048	−0.024	Yes	0.027	Yes
ridgecv	TS	MSC Only	paired *t*-test	−4.47	0.011	−0.149	−3.37	−0.241	−0.056	Yes	0.266	No
ridgecv	TS	SNV + SG	paired *t*-test	−4.26	0.013	−0.149	−2.91	−0.247	−0.052	Yes	0.314	No
ridgecv	TS	SNV	paired *t*-test	−5.60	0.005	−0.195	−3.57	−0.291	−0.098	Yes	0.120	No
ridgecv	True Protein	MSC Only	paired *t*-test	−15.71	0.000	−0.062	−2.33	−0.073	−0.051	Yes	0.002	Yes
ridgecv	True Protein	SNV + SG	paired *t*-test	−6.48	0.003	−0.076	−4.04	−0.109	−0.043	Yes	0.070	No
ridgecv	True Protein	SNV	paired *t*-test	−12.20	0.000	−0.064	−2.41	−0.079	−0.050	Yes	0.006	Yes

## References

[1] Cimmino F., Catapano A., Petrella L., Villano I., Tudisco R., Cavaliere G. (2023). Role of milk micronutrients in human health. Front. Biosci.-Landmark.

[2] Pereira P.C. (2014). Milk nutritional composition and its role in human health. Nutrition.

[3] Etheridge R., Pesti G., Foster E. (1998). A comparison of nitrogen values obtained utilizing the Kjeldahl nitrogen and Dumas combustion methodologies (Leco CNS 2000) on samples typical of an animal nutrition analytical laboratory. Anim. Feed Sci. Technol..

[4] Kleyn D.H., Lynch J.M., Barbano D.M., Bloom M.J., Mitchell M.W. (2001). Determination of fat in raw and processed milks by the Gerber method: Collaborative study. J. AOAC Int..

[5] Stefanov I., Vlaeminck B., Fievez V. (2010). A novel procedure for routine milk fat extraction based on dichloromethane. J. Food Compos. Anal..

[6] Caprita R., Caprita A., Cretescu I. (2014). Determination of lactose concentration in milk serum by refractometry and polarimetry. Sci. Pap. Anim. Sci. Biotechnol..

[7] Poitevin E. (2016). Official methods for the determination of minerals and trace elements in infant formula and milk products: A Review. J. AOAC Int..

[8] Babatunde H.A., Collins J., Lukman R., Saxton R., Andersen T., McDougal O.M. (2024). SVR chemometrics to quantify *β*-lactoglobulin and *α*-lactalbumin in milk using MIR. Foods.

[9] Pegu K., Arya S.S. (2023). Non-thermal processing of milk: Principles, mechanisms and effect on milk components. J. Agric. Food Res..

[10] Stratakos A.C., Inguglia E.S., Linton M., Tollerton J., Murphy L., Corcionivoschi N., Koidis A., Tiwari B.K. (2019). Effect of high pressure processing on the safety, shelf life and quality of raw milk. Innov. Food Sci. Emerg. Technol..

[11] Verruck S., Sartor S., Marenda F.B., da Silva Barros E.L., Camelo-Silva C., Canella M.M., Prudencio E.S. (2019). Influence of heat treatment and microfiltration on the milk proteins properties. Adv. Food Technol. Nutr. Sci..

[12] Cavalcanti R.N., Balthazar C.F., Margalho L.P., Freitas M.Q., Sant’Ana A.S., Cruz A.G. (2023). Pulsed electric field-based technology for microbial inactivation in milk and dairy products. Curr. Opin. Food Sci..

[13] Delorme M.M., Guimarães J.T., Coutinho N.M., Balthazar C.F., Rocha R.S., Silva R., Margalho L.P., Pimentel T.C., Silva M.C., Freitas M.Q. (2020). Ultraviolet radiation: An interesting technology to preserve quality and safety of milk and dairy foods. Trends Food Sci. Technol..

[14] Atik A., Gumus T. (2021). The effect of different doses of UV-C treatment on microbiological quality of bovine milk. LWT.

[15] Qi P.X., Ren D., Xiao Y., Tomasula P.M. (2015). Effect of homogenization and pasteurization on the structure and stability of whey protein in milk. J. Dairy Sci..

[16] Zappi A., Marassi V., Giordani S., Kassouf N., Roda B., Zattoni A., Reschiglian P., Melucci D. (2023). Extracting information and enhancing the quality of separation data: A review on chemometrics-assisted analysis of volatile, soluble and colloidal samples. Chemosensors.

[17] Saxton R., McDougal O.M. (2021). Whey protein powder analysis by mid-infrared spectroscopy. Foods.

[18] Mohamed H., Nagy P., Agbaba J., Kamal-Eldin A. (2021). Use of near and mid infra-red spectroscopy for analysis of protein, fat, lactose and total solids in raw cow and camel milk. Food Chem..

[19] Etzion Y., Linker R., Cogan U., Shmulevich I. (2004). Determination of protein concentration in raw milk by mid-infrared Fourier transform infrared/attenuated total reflectance spectroscopy. J. Dairy Sci..

[20] De Luca M., Ioele G., Spatari C., Caruso L., Galasso M.P., Ragno G. (2019). Evaluation of human breastmilk adulteration by combining Fourier transform infrared spectroscopy and partial least square modeling. Food Sci. Nutr..

[21] Rinnan Å., Van Den Berg F., Engelsen S.B. (2009). Review of the most common pre-processing techniques for near-infrared spectra. TrAC Trends Anal. Chem..

[22] Torniainen J., Afara I.O., Prakash M., Sarin J.K., Stenroth L., Töyräs J. (2020). Open-source python module for automated preprocessing of near infrared spectroscopic data. Anal. Chim. Acta.

[23] Schoot M., Kapper C., van Kollenburg G.H., Postma G.J., van Kessel G., Buydens L.M., Jansen J.J. (2020). Investigating the need for preprocessing of near-infrared spectroscopic data as a function of sample size. Chemom. Intell. Lab. Syst..

[24] Engel J., Gerretzen J., Szymańska E., Jansen J.J., Downey G., Blanchet L., Buydens L.M. (2013). Breaking with trends in pre-processing?. TrAC Trends Anal. Chem..

[25] Zhu X., Guo W., Liu D., Kang F. (2018). Determining the fat concentration of fresh raw cow milk using dielectric spectroscopy combined with chemometrics. Food Anal. Methods.

[26] Guo W., Fang L., Liu D., Wang Z. (2015). Determination of soluble solids content and firmness of pears during ripening by using dielectric spectroscopy. Comput. Electron. Agric..

[27] Feng X.D., Su R., Xu N., Wang X.H., Yu A.M., Zhang H.Q., Cao Y.B. (2013). Portable analyzer for rapid analysis of total protein, fat and lactose contents in raw milk measured by non-dispersive short-wave near-infrared spectrometry. Chem. Res. Chin. Univ..

[28] Li X., Feng F., Gao R., Wang L., Qian Y., Li C., Zhou G. (2016). Application of near infrared reflectance (NIR) spectroscopy to identify potential PSE meat. J. Sci. Food Agric..

[29] Pinto P., Anconi A., de Abreu L., Magalhães E., Nunes C. (2021). Strategies to determine lactose in cow milk by mid infrared spectroscopy. J. Food Compos. Anal..

[30] Amsaraj R., Ambade N., Mutturi S. (2021). Variable selection coupled to PLS2, ANN and SVM for simultaneous detection of multiple adulterants in milk using spectral data. Int. Dairy J..

[31] Inón F., Garrigues S., de la Guardia M. (2004). Nutritional parameters of commercially available milk samples by FTIR and chemometric techniques. Anal. Chim. Acta.

[32] Bergstra J., Bardenet R., Bengio Y., Kégl B. (2011). Algorithms for hyper-parameter optimization. Neural Information Processing Systems.

[33] Khater O., Khater A., Al-Nasr A.S., Abozyd S., Mortada B., Sabry Y.M. (2024). Advancing near-infrared spectroscopy: A synergistic approach through Bayesian optimization and model stacking. Spectrochim. Acta Part A Mol. Biomol. Spectrosc..

[34] Mehdizadeh S.A., Noshad M., Hojjati M. (2024). A modified sequential wavenumber selection-discriminant analysis with Bayesian optimization strategy for detection and identification of chia seed oil adulteration using Raman spectroscopy. Talanta.

[35] Kennard R.W., Stone L.A. (1969). Computer Aided Design of Experiments. Technometrics.

[36] Nogueira F. (2014). Bayesian Optimization: Open Source Constrained Global Optimization Tool for Python. https://github.com/bayesian-optimization/BayesianOptimization.

[37] Scikit-Learn Developers (2023). GroupShuffleSplit: Scikit-Learn Documentation. Scikit-Learn. https://scikit-learn.org/stable/modules/generated/sklearn.model_selection.GroupShuffleSplit.html.

[38] Scikit-Learn Developers (2007). LeavePGroupsOut: Scikit-Learn Documentation. Scikit-Learn. https://scikit-learn.org/stable/modules/generated/sklearn.model_selection.LeavePGroupsOut.html.

[39] Barnes R., Dhanoa M.S., Lister S.J. (1989). Standard normal variate transformation and de-trending of near-infrared diffuse reflectance spectra. Appl. Spectrosc..

[40] Martens H., Jensen S., Geladi P. (1983). Multivariate linearity transformation for near-infrared reflectance spectrometry. Proceedings of the Nordic Symposium on Applied Statistics.

[41] Guo Q., Wu W., Massart D. (1999). The robust normal variate transform for pattern recognition with near-infrared data. Anal. Chim. Acta.

[42] Scikit-Learn Developers (2007). Supervised Learning: Scikit-Learn Documentation. Scikit-Learn. https://scikit-learn.org/stable/supervised_learning.html.

[43] SciPy Developers (2023). SciPy: Scientific Library for Python. SciPy. https://docs.scipy.org/doc/scipy/.

[44] Shapiro S.S., Wilk M.B. (1965). An analysis of variance test for normality (complete samples). Biometrika.

[45] Armstrong R.A. (2014). When to use the B onferroni correction. Ophthalmic Physiol. Opt..

[46] Sullivan G.M., Feinn R. (2012). Using effect size—or why the P value is not enough. J. Grad. Med Educ..

[47] Wu D., He Y., Feng S. (2008). Short-wave near-infrared spectroscopy analysis of major compounds in milk powder and wavelength assignment. Anal. Chim. Acta.

[48] Bonfatti V., Di Martino G., Carnier P. (2011). Effectiveness of mid-infrared spectroscopy for the prediction of detailed protein composition and contents of protein genetic variants of individual milk of Simmental cows. J. Dairy Sci..

[49] Vestergaard R.J., Vasava H.B., Aspinall D., Chen S., Gillespie A., Adamchuk V., Biswas A. (2021). Evaluation of optimized preprocessing and modeling algorithms for prediction of soil properties using vis-nir spectroscopy. Sensors.

